# Preservation of bighead carp heads using black pepper essential oil: biogenic amine inhibition and metabolomic insights

**DOI:** 10.1038/s41538-026-00871-y

**Published:** 2026-04-30

**Authors:** Miao Liu, Lin Li, Jianhui Wang, Na Deng, Hui Li, Zhe Chen, Panxianzhi Ni, Xiaoyi Hou, Paul Van der Meeren

**Affiliations:** 1 School of Food Science and Bioengineering, Changsha University of Science and Technology, Changsha, China; 2 Particle & Interfacial Technology Research Group, Faculty of Bioscience Engineering, Ghent University, Ghent, Belgium; 3 Prepared Dishes Modern Industrial College, Changsha University of Science and Technology, Changsha, China

**Keywords:** Biochemistry, Biological techniques, Biotechnology, Microbiology

## Abstract

This study evaluated the efficacy of black pepper essential oil (BPEO) as a natural preservative for bighead carp heads during superchilling storage, with a focus on controlling biogenic amine (BA)-producing bacteria. Six dominant BA-producing strains were isolated and identified. BPEO exhibited measurable in vitro growth-inhibitory effects and reduced detectable amino acid decarboxylase protein levels in certain strains. Compared with controls, BPEO treatment delayed sensory deterioration, reduced total viable counts in fish meat by approximately 1.0 log CFU/g, lowered TVB-N values by about 24% at day 8, and extended shelf life by approximately 2 days. Untargeted metabolomic analysis indicated that BPEO treatment was associated with alterations in BA-related metabolic pathways. Notably, putrescine (PUT) levels at day 10 were significantly lower in the BPEO-treated groups compared with controls (*p* < 0.001). Reduced PUT accumulation was accompanied by changes in ornithine- and proline-related metabolites in fish meat. This study provides integrated microbiological and metabolomic evidence supporting BPEO as a plant-derived preservation strategy to enhance the safety and quality of aquatic products.

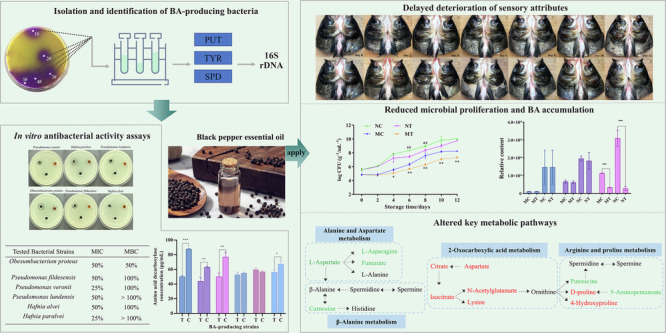

## Introduction

Bighead carp (*Aristichthys nobilis*) is one of China’s four major freshwater fish species, valued for its high nutritional content and desirable flavor. The head of this fish (about 34% of body weight) is especially prized in Chinese cuisine^1^. For example, the renowned Hunan dish “chopped chili fish head” underscores the cultural and culinary importance of bighead carp heads. With increasing demand for ready-to-eat products, fish heads have gained popularity as a nutritious and convenient ingredient. However, their high moisture and nutrient content make carp heads particularly susceptible to microbial growth and spoilage.

Fish spoilage is a complex biochemical process involving both endogenous enzymatic activity and microbial metabolism^2^. Specific spoilage organisms degrade proteins and other nitrogenous substrates, producing biogenic amines (BAs) and other quality-deteriorating compounds through coordinated metabolic pathways. Recent mechanistic and multi-omics studies have highlighted the central role of microbial communities and metabolic process in driving spoilage progression during storage^3,4^. BAs are low-molecular-weight nitrogenous compounds formed by microbial decarboxylation of amino acids in protein-rich foods^5,6^, which are considered important indicators of spoilage in fish products. Excessive BA accumulation is associated with safety concerns and reduced product acceptability.

Although BA formation has been extensively studied in fish fillets^7–9^, comparatively little attention has been given to fish heads. Unlike fillets, fish heads possess a structurally complex matrix characterized by bone-muscle interweaving and matrix heterogeneity. Previous research on bighead carp heads has demonstrated spatial heterogeneity in microbial succession and BA accumulation across different heads compartments, underscoring the distinct spoilage dynamics in this matrix^10^. These anatomical and microbial characteristics suggest that BA accumulation and spoilage progression in fish heads may differ from those in fillets and require targeted investigation.

The surface mucus of fish is a crucial factor in spoilage. While alive, fish mucus acts as a protective barrier with antimicrobial components^11,12^. After death, however, this nutrient-rich mucus (mainly water, proteins, and carbohydrates) becomes an excellent substrate for microbial proliferation^13,14^. For instance, Li et al.^10^ found that *Lactococcus* bacteria in carp head mucus played a critical role in BA generation during superchilling storage. Such findings highlight that controlling microbial activity in the mucus layer is essential to ensure the safety of fish heads.

To ensure food safety, effective preservation strategies for fish heads are needed. Traditional preservation methods like freezing and chilling can slow microbial growth, but they may not fully inhibit BA-producing bacteria over extended storage. In recent years, superchilling storage (−1 to −2 °C) has been increasingly applied in aquatic product preservation, particularly in cold-chain transportation. Compared with conventional chilled conditions, superchilling can more effectively suppress microbial growth and enzymatic activity while minimizing the tissue damage associated with frozen storage^15^. In addition, this temperature range has been previously applied in studies investigating BA formation in bighead carp heads^10^.

Recently, natural antimicrobials such as essential oils have gained attention for improving food safety and shelf life. Essential oils are plant-derived metabolites known for their antibacterial, antifungal, and antioxidant properties^16,17^. Several essential oils, including clove oil (eugenol)^18^, oregano oil (carvacrol)^19,20^, thyme oil (thymol)^19^, and cinnamon oil (cinnamaldehyde)^21^, have been extensively investigated for their antimicrobial activity and ability to reduce BA formation in fish. These oils have been reported to inhibit the formation of putrescine (PUT), cadaverine (CAD), histamine (HIS), tyramine (TYR), and phenylethylamine (PHE), thereby delaying spoilage progression^18–21^. Compared with these commonly studied essential oils, black pepper essential oil (BPEO) extracted from *Piper nigrum* contains diverse bioactive constituents, primarily β-caryophyllene and limonene, along with other monoterpenes such as α-pinene, β-pinene, and sabinene^22–24^. This complex terpenoid profile may contribute to distinct antimicrobial and metabolomic response patterns. Since black pepper is commonly used as a seasoning for fish head dishes, BPEO could offer a dual benefit of flavor and preservation. Although BPEO has demonstrated antimicrobial effects and the ability to extend refrigerated shelf life^25–27^, its efficacy in the structurally complex matrix of bighead carp heads and its specific impact on BA-producing bacteria remain unclear. Importantly, most previous studies have primarily interpreted BA reduction as a consequence of microbial inhibition, while the underlying metabolic pathways associated with BA formation and their modulation by essential oils have received limited attention.

Therefore, this study aimed to evaluate the effects of BPEO on BA-producing bacteria and BA accumulation in bighead carp heads during superchilling storage (−2 °C). Specifically, we (i) isolated and identified dominant BA-producing bacteria from fish heads, (ii) assessed the antimicrobial and BA-suppressing effects of BPEO in vitro and in fish heads, and (iii) applied untargeted metabolomics to characterize metabolite and pathway changes observed under BPEO treatment, particularly those related to BA formation. Accordingly, the study was structured to determine whether reductions in BA accumulation under BPEO treatment are associated with suppression of BA-producing bacteria and decarboxylase-related proteins, and whether such reductions are accompanied by alterations in BA-related metabolites.

## Results and discussion

### Isolation and identification of BA-producing bacteria

Using BA-selective media, 46 bacterial isolates with putative BA-producing ability were obtained from spoiled bighead carp heads (characterized by blue-purple zones around colonies on the indicator medium, Fig. S1). These isolates were categorized based on the amino acid precursor used for screening: 12 from arginine, 5 from ornithine, 4 from phenylalanine, 0 from histidine, 9 from tryptophan, and 16 from lysine selective media. Most isolates were Gram-negative rods forming round, smooth, milky-white colonies (Fig. S2).

All 46 isolates were tested in amino acid decarboxylase broth (Table S1). Nearly all showed arginine and ornithine decarboxylase activities. Moreover, many isolates (including those initially detected on arginine, ornithine, or phenylalanine media) also exhibited lysine decarboxylase activity.

A single BA-producing bacterium can synthesize multiple BAs, although the production levels of specific amines vary significantly^28^. Fourteen representative isolates with strong amino acid decarboxylase activity were selected for HPLC analysis of BA production (Table 1). Among them, strains isolated using phenylalanine, ornithine, and arginine as precursors (P1, P3, O1, A11, A12) synthesized multiple BAs with the exception of histamine. These isolates produced high concentrations of PUT (1025.55–1576.03 μg/mL), CAD (4503.74–4643.50 μg/mL), as well as moderate levels of tryptamine (TRY, 47.10–56.87 μg/mL). In contrast, strains obtained from tryptophan- and lysine-based media produced little or no tryptamine or spermine, possibly due to reduced tryptophan decarboxylase activity under liquid culture conditions. None of the isolates produced histamine, consistent with the absence of histidine-positive strains in the initial screening.

**Table 1:** The biogenic amine production capability of strains isolated from bighead carp heads; histamine was not detected in any of the samples

Strain number	Biogenic amine (μg/mL)						
	Tryptamine	Phenethylamine	Putrescine	Cadaverine	Tyramine	Spermidine	Spermine
P1	55.05 ± 6.57^a^	13.42 ± 1.04^cd^	1066.17 ± 34.87^c^	4528.78 ± 106.90^a^	6.03 ± 0.94^bc^	4.52 ± 0.33^f^	3.40 ± 0.27^a^
P3	47.10 ± 2.25^b^	14.11 ± 0.60^bcd^	1025.55 ± 30.08^c^	4552.48 ± 96.07^a^	7.47 ± 0.60^b^	2.92 ± 0.08^fg^	3.53 ± 0.17^a^
O1	56.87 ± 2.66^a^	14.10 ± 1.25^bcd^	1028.29 ± 17.44^c^	4503.74 ± 64.58^a^	5.62 ± 0.84^bcd^	3.61 ± 0.05^fg^	3.09 ± 0.07^a^
A11	52.01 ± 8.96^ab^	16.85 ± 2.00^b^	1323.90 ± 37.44^b^	4643.50 ± 75.92^a^	5.65 ± 3.58^bcd^	4.37 ± 0.31^f^	3.11 ± 0.80^a^
A12	47.91 ± 1.51^b^	56.48 ± 2.37^a^	1576.03 ± 185.91^a^	4585.91 ± 201.01^a^	5.21 ± 0.19^bcde^	2.12 ± 0.17^g^	2.20 ± 0.12^b^
T3	ND	7.92 ± 2.00^ef^	7.40 ± 0.41^d^	ND	52.21 ± 3.82^a^	15.25 ± 2.82^a^	ND
T4	3.27 ± 0.13^c^	7.58 ± 1.10^f^	1.20 ± 0.21^d^	ND	2.37 ± 0.25^ef^	8.35 ± 0.79^e^	ND
L1	ND	7.94 ± 0.94^ef^	4.53 ± 0.20^d^	ND	1.34 ± 0.07^f^	8.00 ± 0.40^e^	ND
L3	ND	8.39 ± 0.57^ef^	4.40 ± 0.20^d^	0.17 ± 0.14^b^	2.40 ± 0.26^ef^	12.86 ± 0.30^b^	ND
L7	ND	13.57 ± 2.50^bcd^	4.80 ± 0.55^d^	1.39 ± 0.25^b^	3.30 ± 0.12^cdef^	11.92 ± 0.35^bc^	ND
L9	ND	10.99 ± 0.56^de^	4.68 ± 0.45^d^	3.80 ± 0.40^b^	2.80 ± 0.28^def^	9.85 ± 0.79^de^	ND
L10	ND	16.08 ± 0.75^bc^	4.27 ± 0.12^d^	0.38 ± 0.07^b^	2.78 ± 0.48^def^	10.47 ± 0.65^cd^	ND
L12	ND	16.68 ± 0.88^bc^	5.44 ± 0.33^d^	1.87 ± 0.21^b^	2.78 ± 0.37^def^	15.06 ± 0.45^a^	ND
L14	ND	15.13 ± 3.03^bc^	5.01 ± 0.77^d^	0.80 ± 0.13^b^	2.82 ± 0.24^def^	16.48 ± 1.35^a^	ND

Note: Different superscript letters (a–g) in the same column indicate significant differences (*p* < 0.05). “ND” represents “not detected”.

Six dominant BA-producing strains were identified by 16S rDNA sequencing (Table S2). These were *Pseudomonas lundensis* (P3), *Obesumbacterium proteus* (O1), *Hafnia paralvei* (A11), *Hafnia alvei* (A12), *Pseudomonas veronii* (T3, L7), and *Pseudomonas fildesensis* (L3, L12, L14), all displaying sequence similarity greater than 97%. These bacteria are known spoilage organisms and have been associated with BA formation in chilled fish products.

### Antibacterial effect of BPEO on isolated BA-producing bacteria

The inhibition zone diameters of BPEO and pepper oleoresin against the six isolated characteristic BA-producing bacteria are depicted in Table 2. Notably, pepper oleoresin exhibited no antibacterial activity against the characteristic BA-producing strains, whereas BPEO displayed clear inhibitory effects. Compared with *Hafnia* spp., BPEO showed stronger inhibition against *Obesumbacterium proteus* and *Pseudomonas* spp. The inhibition zone diameters of BPEO against *Pseudomonas* spp. ranged from 16.0 to 17.7 mm, without significant differences among the three tested strains. Furthermore, BPEO exhibited a significantly stronger inhibitory effect on *Hafnia alvei* compared to *Hafnia paralvei* (*p* < 0.05).

**Table 2:** Inhibition zone diameter, minimum inhibitory concentration (MIC), and minimum bactericidal concentration (MBC) of black pepper essential oil (BPEO) against six BA-producing bacteria

Tested Bacterial Strains	Inhibition zone diameter (mm)	MIC	MBC
*Obesumbacterium proteus*	17.5 ± 0.8^a^	50%	50%
*Pseudomonas fildesensis*	17.7 ± 0.4^a^	50%	100%
*Pseudomonas veronii*	16.9 ± 0.4^ab^	25%	100%
*Pseudomonas lundensis*	16.0 ± 0.4^b^	50%	>100%
*Hafnia alvei*	14.7 ± 0.2^c^	50%	100%
*Hafnia paralvei*	11.5 ± 0.4^d^	25%	>100%

Note: Different superscript letters (a–d) indicate significant differences in the diameter of inhibitory zones (*p* < 0.05). A pure essential oil sample is 100% concentrated, while “>100%” indicates that no bacteriostatic ability was detected for pure essential oil.

The effects of BPEO on the MIC and MBC values of the six BA-producing strains are shown in Table 2. BPEO exhibited varying degrees of inhibitory abilities against the six strains, with MIC values ranging from 25 to 50%, while bactericidal effects were limited under the conditions tested. Based on MIC values, *Pseudomonas veronii* and *Hafnia paralvei* were relatively more sensitive to BPEO, which was different from the inhibition zone assay results. This discrepancy may be attributed to differences in diffusion capacity and solubility of the hydrophobic terpenoid constituents of BPEO under different culture media conditions.

BPEO is widely reported to be rich in terpenoid compounds, particularly β-caryophyllene and limonene, which have been documented to exhibit bacteriostatic activity^29,30^. Owing to their hydrophobic nature, they can partition into bacterial cell membranes, disrupting lipid bilayer integrity and increasing membrane permeability^31^. Among these, β-caryophyllene has been demonstrated to exert antimicrobial activity primarily through membrane-targeting mechanisms, including alteration of membrane permeability, destabilization of lipid bilayers, and induction of intracellular content leakage^32,33^. Such membrane perturbation may interfere with the synthesis of critical macromolecules, such as DNA, RNA, proteins, and polysaccharides, thereby impairing bacterial viability^34,35^. Therefore, the inhibitory activity of BPEO against the six BA-producing strains may be associated with the terpenoid-rich composition. Nevertheless, the translation of these in vitro results to real food systems requires consideration of practical dosage, sensory acceptability, and compliance with applicable safety regulations.

### Amino acid decarboxylase levels

**Fig. 1:**
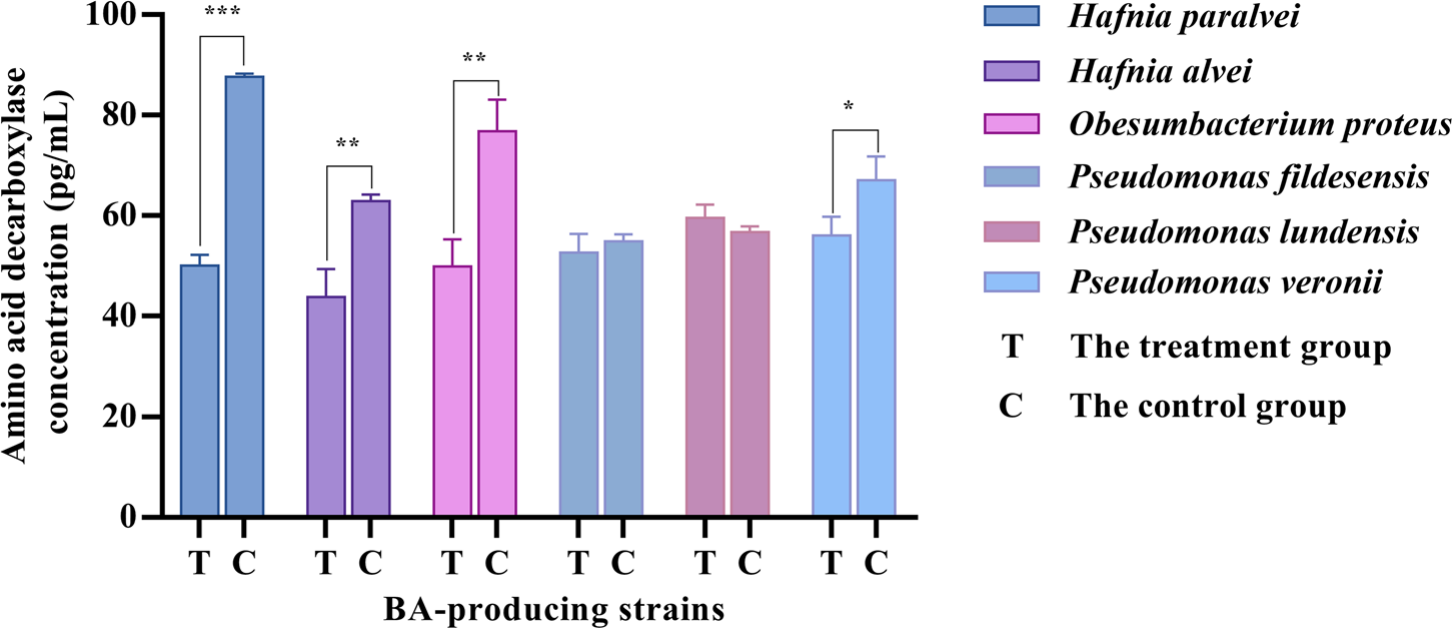
Effect of BPEO treatment (T) compared with control (C) on amino acid decarboxylase protein levels in six BA-producing strains. Data are presented as mean ± SD (*n* = 6). Statistical analysis was performed using one-way ANOVA followed by Duncan’s multiple range test. *p < 0.05, **p < 0.01, ***p < 0.001.

BAs are formed by microorganisms through the action of decarboxylases. These enzymes specifically target certain amino acids, removing their carboxyl groups to produce the corresponding amines and release CO₂^36^. To evaluate the influence of BPEO on decarboxylase-related protein expression, amino acid decarboxylase protein levels of six characteristic BA-producing bacteria were determined and illustrated in Fig. 1. Compared with the corresponding control groups, four of the six strains exhibited significantly lower amino acid decarboxylase levels following BPEO treatment, whereas *Pseudomonas lundensis* and *Pseudomonas fildesensis* showed no significant differences. These results indicate that BPEO treatment was associated with reduced detectable decarboxylase-related protein abundance in certain strains.

Previous studies have reported that certain essential oils can modulate microbial decarboxylation-related pathways at the gene or protein level. For example, Li et al. demonstrated that *Coreopsis tinctoria* essential oil significantly inhibited the growth of microorganisms and suppressed the expression of *tyr DC* and *tyr P* genes in the tyrosine decarboxylation pathway (*p* < 0.05), thereby reducing tyrosine accumulation^37^. Taken together, these results suggest that reduction in amino acid decarboxylase protein levels may represent one potential pathway-level association contributing to reduced BA accumulation. However, as enzymatic catalytic activity was not directly measured in the present study, further investigation is required to clarify the precise molecular mechanism.

### Inhibitory effect of BPEO on the BA formation in bighead carp heads

**Fig. 2:**
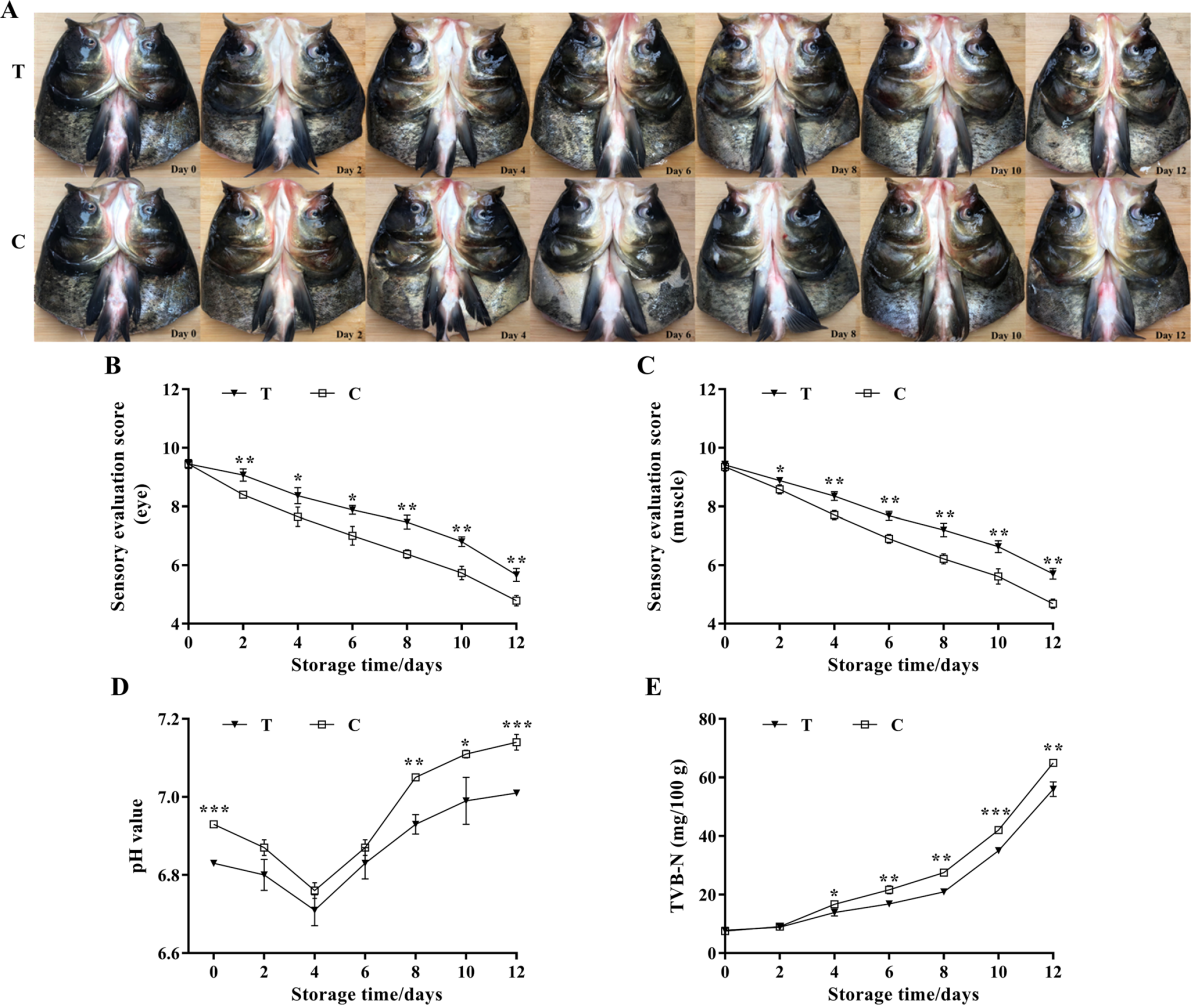
Effect of BPEO treatment on quality indicators of bighead carp heads during superchilling storage. **A** Representative images of bighead carp heads during storage. **B**, **C** Sensory evaluation scores for eye appearance and muscle quality. **D** Changes in pH. **E** Changes in total volatile base nitrogen (TVB-N). Data are presented as mean ± SD (*n* = 3). Statistical analysis was performed using one-way ANOVA followed by Duncan’s multiple range test. *p < 0.05, **p < 0.01, ***p < 0.001 indicate significant differences between treatment and control groups at the same time point.

The changes in eye appearance and muscle quality of bighead carp heads during storage are illustrated in Fig. 2A. On day 0, both groups exhibited intact and elastic eyeballs and firm muscle tissue, indicating high initial sensory quality. As storage progressed, the control group showed pronounced deterioration in eye appearance, characterized by opaque corneas, hemorrhaging in the irises and eye sockets, as well as progressive softening of muscle tissue, reduced water-holding capacity, and the development of unpleasant fishy odor. These changes were accompanied by a significant decline in both eye appearance and muscle quality scores over time. In contrast, the BPEO-treated group exhibited a slower deterioration of sensory attributes. Compared with the control group, BPEO-treated samples maintained higher eye appearance scores and muscle quality scores at corresponding storage times (Fig. 2B, C). These findings are consistent with previous studies on muscle-based food matrices (e.g., fish fillets and meat products)^38^, whereas the present study focuses on fish heads, which represent a distinct anatomical and microbial system.

Figure 2D shows the pH evolution in fish heads during storage. On day 0, the control group displayed an average pH of 6.93, whereas the treatment group exhibited a significantly lower pH value (*p* < 0.001). This may be attributed to the intrinsic acidity of BPEO, which contributed to an overall pH reduction post-treatment. Overall, both groups showed a similar trend over time, with an initial decline in pH followed by a gradual increase, which was consistent with previous studies on bighead carp fillets^8,9^. During the early storage period (days 0–4), the decrease in pH is commonly associated with the accumulation of acidic metabolites, such as lactic acid derived from glycogen breakdown and pyrophosphate generated during ATP decomposition^39^. In the later stages (days 4–12), however, protein degradation and microbial metabolism led to the accumulation of alkaline nitrogenous compounds, including BAs and ammonia, which are widely recognized contributors to the gradual increase in pH during fish spoilage^40^. Although amino acid decarboxylation releases CO_2_, its contribution to pH changes cannot be clearly distinguished under the present conditions. Therefore, the discussion of pH evolution focuses on alkaline nitrogenous metabolites, as reflected by BAs and TVB-N measured in this study. Throughout the whole storage period, the treatment group maintained a consistently lower pH compared to the control group, with significant differences emerging on days 8, 10 and 12 (*p* < 0.05). This pattern may be associated with reduced microbial proliferation and altered amino acid metabolism under BPEO treatment, potentially limiting the accumulation of alkaline compounds.

TVB-N is widely used as an indicator of protein degradation and fish spoilage. It reflects the accumulation of nitrogenous compounds, such as ammonia and amines, generated through enzymatic reactions and microbial metabolism during storage^41^. The changes in TVB-N during superchilling storage of bighead carp heads are depicted in Fig. 2E. In both groups, TVB-N values increased progressively over time, with the values in the treatment group significantly lower than those of the control group (*p* < 0.05). According to the Chinese national standard for freshwater fish (GB 2733-2015), the acceptable limit of TVB-N is 20 mg/100 g. Exceeding this limit is generally considered indicative loss of freshness and reduced consumer acceptability. In the present study, the control group exceeded this limit at day 6 (21.59 ± 1.40 mg/100 g), whereas the BPEO-treated group reached comparable levels only at day 8 (20.92 ± 1.10 mg/100 g). TVB-N levels in the treated group were approximately 24% lower than in the control group at day 8. These results suggest that BPEO delayed the increase of TVB-N during storage, thereby delaying the progression toward regulatory spoilage limits under the tested conditions. Similarly, Huang et al.^42^ observed that essential oils treatment delayed the increase of TVB-N and PUT levels in grass carp fillets during chilled storage.

**Fig. 3:**
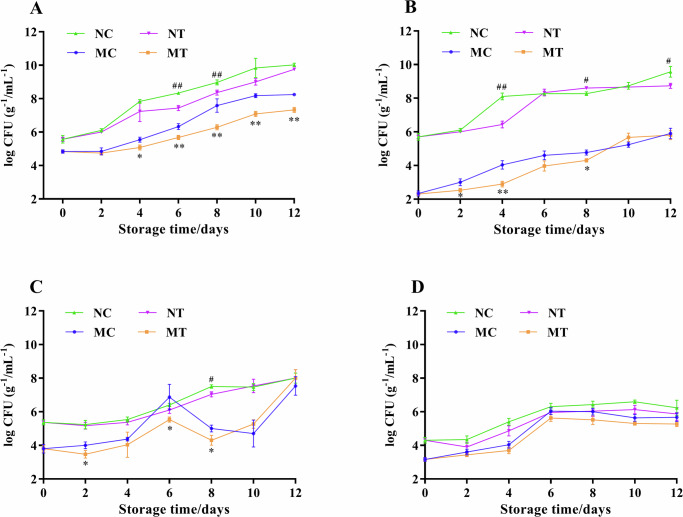
Effect of BPEO treatment on microbial counts during superchilling storage. **A** Total viable counts (TVC), **B** *Pseudomonas* counts, **C** H_2_S-producing bacteria counts, and **D** *Aeromonas* counts in bighead carp head muscle (M) and surface mucus (N). Data are presented as mean ± SD (*n* = 3). Statistical analysis was performed using one-way ANOVA followed by Duncan’s multiple range test. *p < 0.05, **p < 0.01 indicate significant differences between MC and MT at the same time point; #*p* < 0.05, ##*p* < 0.01 indicate significant differences between NC and NT at the same time point.

The effects of BPEO treatment on microorganisms in bighead carp heads and surface mucus during superchilling storage are shown in Fig. 3. Overall, the total viable count (TVC) increased progressively in all four samples during storage. However, significantly lower TVC values were observed in BPEO-treated samples at specific storage time points (*p* < 0.05), indicating a slower increase in microbial load compared with the control (Fig. 3A). In the control group, the TVC of the fish meat and mucus increased from 4.84 ± 0.26 log CFU/g and 5.57 ± 0.34 log CFU/mL on day 0 to 8.24 ± 0.24 log CFU/g and 10.02 ± 0.32 log CFU/mL, respectively. In contrast, BPEO-treated samples displayed corresponding final TVC levels of 7.33 ± 0.28 log CFU/g and 9.75 ± 0.22 log CFU/mL, representing approximately a 1 log unit reduction (Fig. 3A). According to the International Commission on Microbiological Specifications for Foods (ICMSF), 7log CFU/g is commonly considered an upper limit for acceptable microbial quality in fish^43^. In the present study, the TVC of the BPEO-treated fish meat remained below this threshold through day 8, whereas the control group exceeded it earlier. These results suggest that BPEO treatment was associated with a reduced rate of microbial proliferation during superchilling storage of fish heads.

Among the six BA-producing bacteria identified, three strains were classified as *Pseudomonas* spp. This indicates that *Pseudomonas* may represent an important BA-producing group during the superchilling storage of bighead carp heads. From day 0 to day 8, *Pseudomonas* counts showed a similar trend as that of the corresponding TVC results. However, the *Pseudomonas* count in the BPEO-treated fish meat increased rapidly after day 8, reaching levels comparable to or slightly higher than those in the control group by day 10 (Fig. 3B). This change may be associated with a reduction in the effective concentration of BPEO in the later stages of storage, possibly influenced by physicochemical factors such as volatility and diffusion^44^. As essential oils are widely reported to interact with bacterial membranes through terpenoid constituents, a reduction in active components may have attenuated the inhibitory effect over time^45^. Moreover, *Pseudomonas* counts in mucus samples were significantly higher than those in the meat (*p* < 0.05), reflecting distinct microbial distributions between tissue types under the present storage conditions.

H_2_S-producing bacteria, an important component of the microbial flora in bighead carp heads, are associated with the development of off-odors. As storage time progressed, the number of H_2_S-producing bacteria in both fish meat and mucus gradually increased, with higher counts in the mucus than in the meat, following a trend similar to that of TVC and *Pseudomonas* (Fig. 3C). Although BPEO did not consistently suppress H_2_S-producing bacteria across the entire storage period, it resulted in a significant reduction compared with the control on day 8 (*p* < 0.05). *Aeromonas*, a common spoilage-associated genus in freshwater fish, increased during the early storage period (days 0–6), after which growth stabilized or slightly declined (Fig. 3D). No significantly inhibitory effect of BPEO on *Aeromonas* proliferation was observed. Taken together, these findings suggest that BPEO exhibited selective effects on microbial groups, with more evident influence on TVC and *Pseudomonas*-associated dynamics, while exerting limited inhibition against H₂S-producing bacteria and *Aeromonas*. Considering both the TVB-N regulatory threshold and microbial limit, BPEO treatment delayed the time required to reach these spoilage criteria by approximately 2 days under the tested superchilling conditions.

### Metabolomics analysis of BPEO’s impact on BA formation

**Fig. 4:**
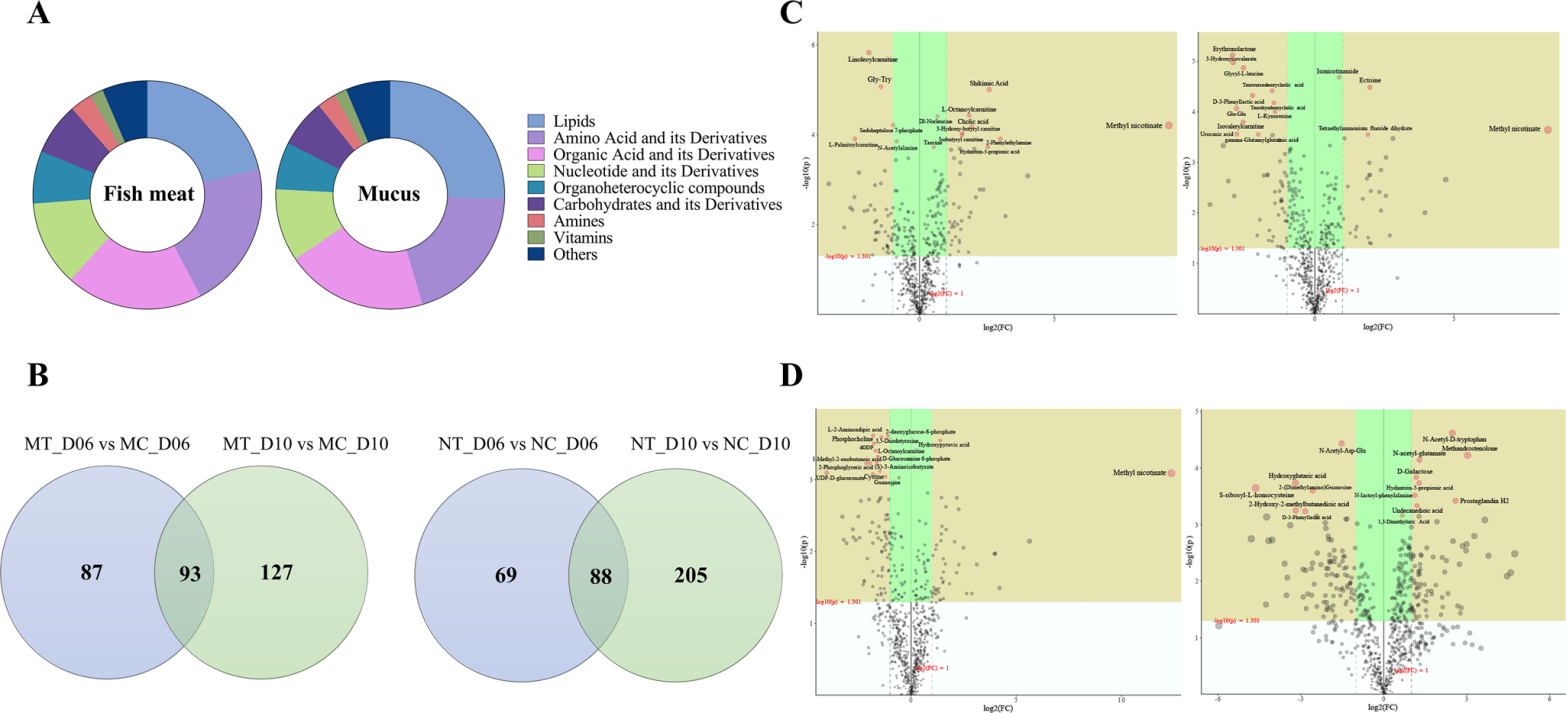
Effect of BPEO treatment on metabolomic profiles of fish meat and surface mucus during superchilling storage. **A** Metabolite classification for fish meat and mucus samples. **B** Venn diagram showing overlapping metabolites at different storage times. **C** Volcano plot of differential metabolites in fish meat at day 6 and day 10. **D** Volcanic plot of differential metabolites in surface mucus at day 6 and day 10.

Metabolites identified in fish meat and mucus samples are summarized in Fig. 4A. A total of 702 and 748 metabolites were identified in fish meat and mucus samples, respectively. The metabolite categories and their relative abundances showed notable consistency between the two groups. The top three categories, comprising lipids, amino acids and their derivatives, and organic acids and their derivatives, accounted for 61.7–65.5% of the total metabolites. Orthogonal partial least squares discriminant analysis (OPLS-DA) demonstrated clearly separation between BPEO-treated and control samples. The *Q*² values for fish meat (0.926) and mucus (0.893) indicated strong model reliability and predictive ability, suggesting that BPEO treatment was associated with significant alterations in metabolic profiles (Fig. S3).

Differential metabolites were screened based on their variable importance in projection (VIP > 1) and statistical significance (*p* < 0.05). Compared with controls, 180 and 220 differential metabolites were identified in fish meat on days 6 and 10, respectively, with 93 shared metabolites. Mucus samples showed 157 and 293 differential metabolites on days 6 and 10, with 88 shared (Fig. 4B). Volcano plots (Fig. 4C, D) were generated to visualize the distribution of these differential metabolites according to fold change and statistical significance. For clarity, the top 15 ranked differential metabolites were highlighted in red as representative features.

Comprehensive data on the temporal dynamics of these shared differential metabolites in fish meat and mucus samples under BPEO treatment are provided in Tables S3 and S4, respectively. Concretely, 93 shared differential metabolites were identified in fish meat samples, encompassing major classes such as carbohydrates (20 metabolites), lipids (14 metabolites), nucleic acids (8 metabolites), steroids (8 metabolites), peptides (6 metabolites), vitamins and cofactors (6 metabolites), and organic acids (4 metabolites). The majority of these metabolites showed a downregulation trend, aligning with observations from the volcano plots. Notably, carbohydrates exhibited a sharper decline by day 10, whereas peptides, vitamins, and cofactors displayed significant downregulation as early as day 6. In the mucus samples, 88 shared differential metabolites were detected, distributed across some key classes including carbohydrates (18 metabolites), lipids (10 metabolites), peptides (10 metabolites), organic acids (8 metabolites), steroids (8 metabolites), nucleic acids (4 metabolites), as well as vitamins and cofactors (4 metabolites).

KEGG enrichment analysis was conducted separately for significantly differential metabolites identified in fish meat and mucus groups, with a focus on pathways achieving statistical significance (*p* < 0.05). The principal enriched pathways were visualized in Fig. S4. In both fish meat and mucus samples, the most significantly enriched pathways were primarily associated with biosynthesis of amino acids, nucleotide metabolism, and central carbon metabolism-related processes. Although the relative ranking of individual pathways differed between matrices, the enrichment profiles showed substantial overlap in core metabolic categories, suggesting that BPEO intervention influenced core metabolic networks across different tissue environments.

**Fig. 5:**
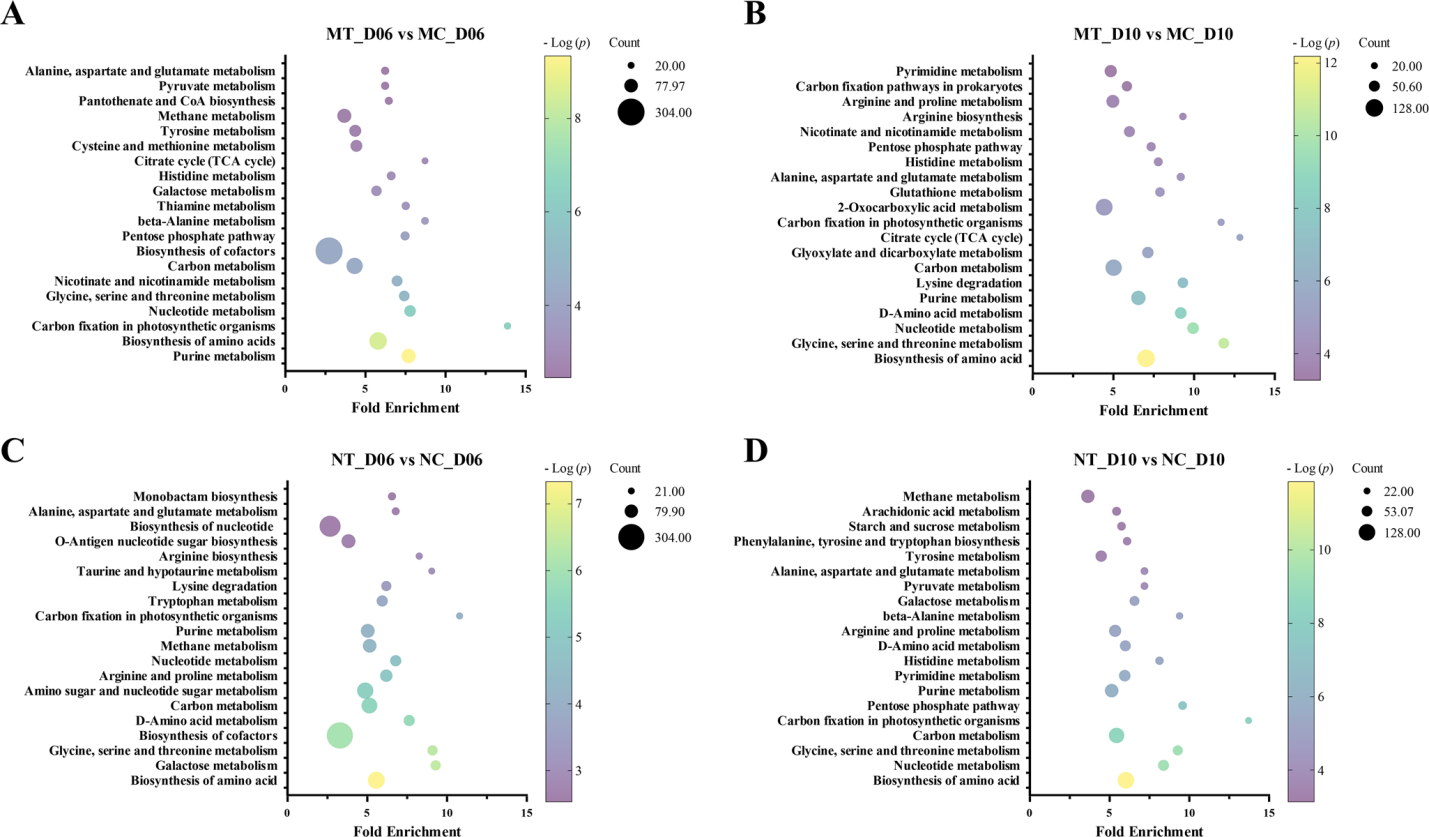
Enrichment analysis of metabolic pathways following BPEO treatment. Bubble plots of significantly enriched metabolic pathways in fish meat (**A**, **B**) and mucus samples (**C**, **D**) following BPEO treatment. **A**, **C** correspond to day 6, whereas **B**, **D** correspond to day 10. Bubble size represents the number of differential metabolites mapped to each pathway, and color indicates the enrichment significance.

Bubble plots illustrating the dominant differential metabolic pathways in fish meat and mucus samples at different storage times following BPEO treatment are presented in Fig. 5. Notably, biosynthesis of amino acids was consistently enriched across both time points and matrices. Given that free amino acid accumulation during fish spoilage is closely linked with proteolysis and microbial metabolism, this enrichment pattern indicates that BPEO treatment was associated with broader changes in nitrogen-related metabolism rather than selective effects on a single decarboxylation pathway. In parallel, central carbon metabolism was repeatedly enriched, including carbon metabolism, pyruvate metabolism, the TCA cycle, and the pentose phosphate pathway. These pathways constitute the core of cellular energy generation and biosynthetic precursor supply. Similarly, enrichment of nucleotide metabolism may reflect changes in growth-related biosynthetic demand, consistent with the observed growth-delaying effects during storage.

In mucus samples, enrichment of D-amino acid metabolism and amino sugar-related pathways was observed, accompanied by increased levels of lysophosphatidylcholine (e.g., Lyso-PC 16:0 and Lyso-PC 18:1) at day 10. These changes may indicate adjustments in cell envelope-associated metabolic processes in microbial communities directly exposed to BPEO. These alterations may be discussed in the context of membrane-targeting properties reported for the terpenoid constituents of BPEO, particularly β-caryophyllene^33,45^. Although the present metabolic data do not directly establish causality, the repeated enrichment of central carbon, nitrogen, and nucleotide metabolism across both fish and mucus samples supports the interpretation that BPEO treatment was accompanied by multi-level metabolic adjustments.

**Fig. 6:**
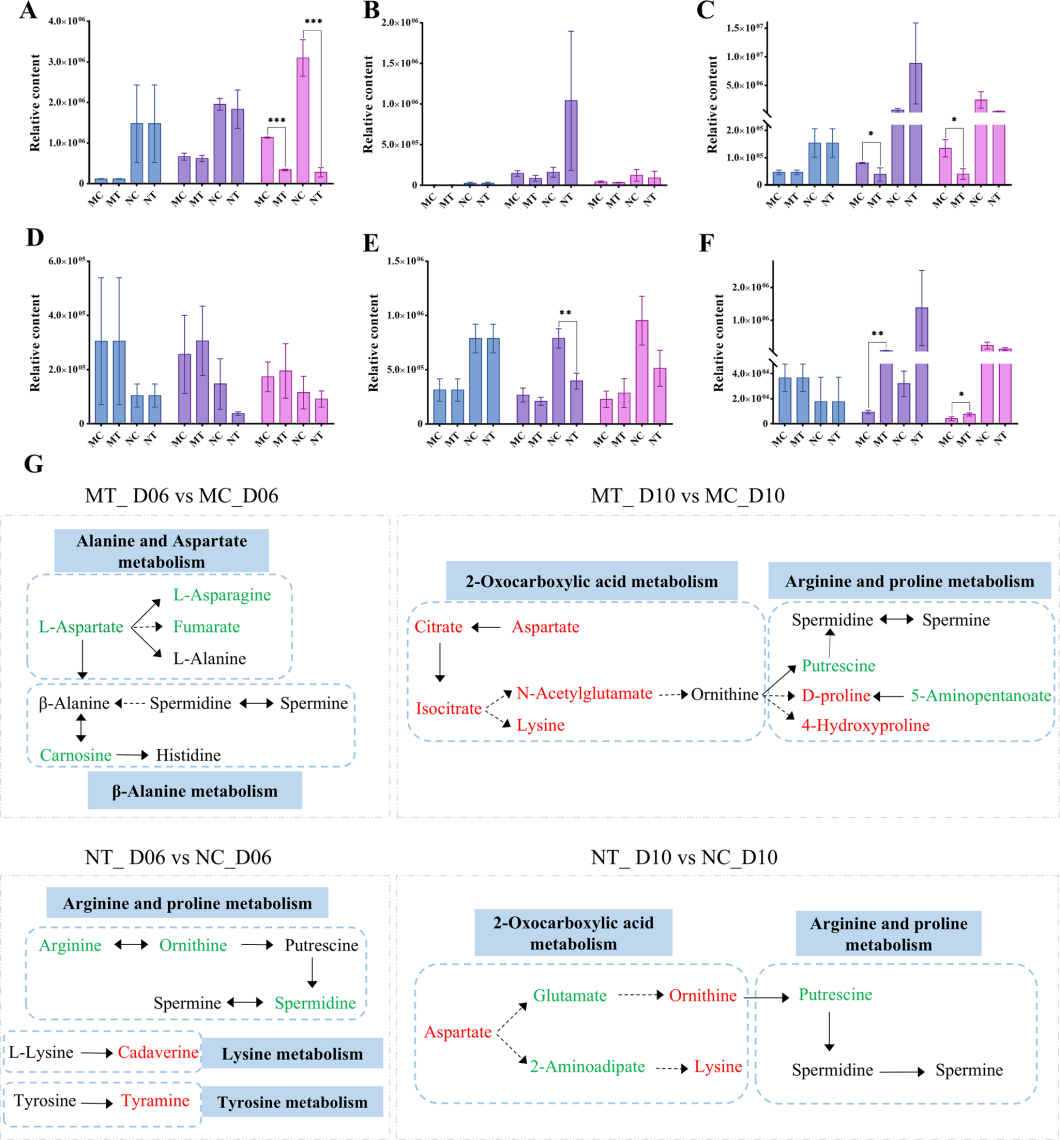
Changes in BA contents and associated metabolic pathways following BPEO treatment. Relative contents of BAs, including **A** putrescine (PUT), **B** cadaverine (CAD), **C** tyramine (TYR), **D** spermine (SPM), **E** spermidine (SPD), **F** phenylethylamine (PHE), in fish meat and mucus samples. **G** Proposed integrative schematic summarizing associations between BPEO treatment and BA-related metabolic alterations. Metabolites showing significant increases are highlighted in red, whereas those with significant decreases are marked in green. Solid arrows represent established biochemical reactions, and dashed arrows denote associations inferred from metabolomic analysis. Data are presented as mean ± SD (*n* = 3). Statistical analysis was performed using one-way ANOVA followed by Duncan’s multiple range test. *p < 0.05, **p < 0.01, ***p < 0.001.

The relative concentrations of BAs in fish meat and mucus samples following BPEO treatment are shown in Fig. 6A–F. Six BAs were detected, namely PUT, CAD, TYR, spermine (SPM), spermidine (SPD), and PHE. Among these, four BAs showed significant changes after BPEO treatment. Notably, PUT levels were significantly decreased on day 10 in both fish meat and mucus groups (*p* < 0.001). TYR showed a significant decrease in fish meat group (*p* < 0.05), whereas SPD was significantly reduced only in the mucus group (*p* < 0.01). Despite the differential responses among individual BAs, BPEO treatment suppressed the accumulation of major spoilage-related BAs, particularly PUT.

KEGG pathway mapping highlighted several BA-related metabolic pathways, including alanine and aspartate metabolism, β-alanine metabolism, 2-oxocarboxylic acid metabolism, arginine and proline metabolism, lysine degradation, and tyrosine metabolism, that were associated with differential metabolites following BPEO treatment. Based on these results, an illustrative schematic diagram (Fig. 6G) was constructed to visualize these pathway-level associations. In fish meat samples, multiple amino acid-related metabolites differed between groups (Fig. S5). At day 6, these amino acid-related changes were not accompanied by a significant reduction in BA levels. By day 10, PUT levels declined significantly, coinciding with increased relative levels of d-proline and 4-hydroxyproline. As ornithine is upstream of PUT formation and is metabolically linked to proline-related pathways, this pattern may reflect a shift in ornithine-related metabolism under BPEO treatment. In mucus samples, CAD and TYR levels increased at day 6, whereas SPD levels decreased, suggesting that BA responses to BPEO were not uniform across classes and may vary with storage stage. By day 10, relative levels of ornithine and lysine were elevated, whereas PUT levels declined significantly. The coexistence of increased precursor availability (e.g., ornithine) and reduced PUT accumulation suggests that the conversion step from ornithine to PUT may have been limited under BPEO treatment. This observation coincides with the reduced detectable amino acid decarboxylase protein levels observed in certain strains, supporting an association between alterations in decarboxylase-associated processes and decreased PUT formation in mucus samples. A simplified schematic summarizing the proposed associations between BPEO treatment, microbial suppression, decarboxylase-related protein reduction, and BA-associated metabolite alterations is presented in Fig. S6.

Comparative analysis of BA-related metabolic pathways in fish meat and mucus samples indicates that BPEO was associated with reduced PUT accumulation through matrix-specific metabolic adjustments. These associations involve alterations in ornithine-related metabolism in fish meat and reduced decarboxylase-associated protein levels detected in isolated BA-producing strains. However, as untargeted metabolomic profiling provides correlative evidence of metabolic alteration and does not directly measure enzymatic activity or metabolic flux, these interpretations should be regarded as associative rather than definitive mechanism confirmation. Moreover, the metabolomic profiles likely reflect combined host-microbial responses and do not establish direct causality. Targeted enzymatic and multi-omics analyses are required to confirm specific regulatory mechanisms.

Although BPEO demonstrated antimicrobial and metabolic effects under superchilling conditions, a reduction in preservative efficacy was observed during late storage stages. This reduction in efficacy may be related to the inherent volatility of essential oil constituents, leading to gradual loss of active components over time. In addition, microbial adaptation to sublethal essential oil exposure may contribute to the reduced inhibitory efficacy during prolonged storage. Future studies should focus on improving the stability and sustained release of BPEO in complex food matrices, for example through encapsulation approaches, controlled-release systems, or integration into packaging materials.

This study demonstrates that BPEO can function as a plant-derived preservative strategy for bighead carp heads under superchilling conditions by delaying microbial proliferation and modulating BA-related metabolic patterns. Integrated microbiological and metabolomic analyses indicate that BPEO treatment was associated with reduced PUT accumulation and alterations in decarboxylase-related processes. However, the preservative efficacy of BPEO diminished during late storage stages, possibly due to the volatility of essential oil constituents and microbial adaptation. Moreover, the mechanistic interpretations derived from untargeted metabolomics remain correlative and require further targeted validation. Future studies should focus on improving the stability and sustained release of BPEO in complex matrices and evaluating its performance under industrial processing conditions. Overall, this work provides a scientific foundation for applying plant-based essential oils to enhance the safety, quality and storage stability of aquatic products.

## Methods

### Materials and reagents

BPEO was obtained from Anhua Jinhou Biological Technology Co., Ltd. (Hunan Province, China). Microbiological media including tryptic soy agar (TSA), plate count agar, iron agar medium, Luria-Bertani (LB) broth, *Pseudomonas* CFC selective agar and *Aeromonas* ampicillin selective agar were purchased from commercial suppliers (Guangdong Huankai and Qingdao Hope, China). All other reagents were supplied by Shanghai Aladdin Biochemical Technology Co., Ltd. (Shanghai, China) and Sinopharm Chemical Reagent Co., Ltd. (Shanghai, China).

### Bighead carp sample preparation

Fresh bighead carp (approximately 1.5 kg each) was purchased from a local market in Changsha, China. Live fish were transported to the laboratory and immediately processed; gills were removed and the fish were cleaned. Heads were excised 5 cm below the gill arch, bisected, gently rinsed with sterile distilled water to remove visible residual blood, and drained. The natural surface mucus layer remained on the fish head surface. The prepared heads were divided into two independent groups for different experimental purpose. One group, used exclusively for the isolation of BA-producing bacteria, was packaged in sterile bags and stored at −2 °C. The other group was used to evaluate the preservative effect of BPEO during storage. In this group, fish heads in the treatment group were uniformly sprayed with BPEO using a 10-mL fine-mist sprayer, applying 2 mL of BPEO to each fish head, while the control heads were sprayed with an equal volume of sterile water. The 2 mL application volume was selected based on preliminary dose-screening to ensure uniform coverage and sensory acceptability. After spraying, samples were drained, then individually sealed in sterile bags and stored at −2 °C for 12 days. Quality indices were measured every 2 days.

### Isolation and identification of BA-producing bacteria

BA-producing bacteria were screened using a BA indicator agar medium as described previously with minor modifications^46,47^. The BA indicator agar medium consisted of peptone (0.5%), yeast extract (0.5%), NaCl (0.5%), agar (2.0%), bromocresol purple (0.012%), and a single amino acid precursor (1.0%; phenylalanine, histidine, arginine, ornithine, lysine, or tryptophan). The medium was sterilized and then adjusted to pH 5.0 ± 0.2 under aseptic conditions. Muscle tissue and mucus from stored fish heads were collected at day 12 (−2 °C), corresponding to the late storage stage when BA levels have been reported to increase under similar superchilling conditions^10^. Samples were homogenized and serially diluted. Aliquots were spread onto the BA indicator agar and incubated at 30 °C for 48 h. Colonies exhibiting a blue or purple halo were considered presumptive BA producers and were picked and restreaked on TSA for purification. Purified isolates were inoculated into amino acid decarboxylase broth containing peptone (0.5%), yeast extract (0.5%), glucose (0.1%), pyridoxal phosphate (0.005%), bromocresol purple (0.012%), and the corresponding amino acid (0.1%), with the pH adjusted to 5.5 ± 0.2. After incubation at 30 °C for 48 h, a color change of bromocresol purple from purple to yellow indicated amino acid decarboxylase activity, confirming the BA-producing potential of the isolates.

Selected isolates were tested for actual BA production using high-performance liquid chromatography (HPLC). Each strain was first grown in LB broth (30 °C, 12 h). 0.2 mL of this culture was transferred to 10 mL of LB containing 0.1% of a specific amino acid and 0.01% pyridoxal phosphate. After shaking at 30 °C for 48 h, 1 mL of culture was mixed with 1 mL of 5% trichloroacetic acid (TCA), and centrifuged (12,000 rpm, 10 min, 4 °C). The supernatant was analyzed by HPLC following Li et al.^10^ to identify and quantify BAs produced.

Bacterial DNA was extracted using the TIANamp Bacteria DNA Kit. Primers 27 F (5’-AGAGTTTGATCCTGGCTCAG-3’) and 1492 R (5′-CTACGGCTACCTTGTTACGA-3′) were used for PCR amplification (Table S5). PCR products were purified using the AxyPrep DNA Gel Extraction Kit and sequenced on the ABI 3730-XL sequencer (Applied Biosystems, USA). The assembled sequences were compared with the NCBI 16S rDNA database using the NCBI BLAST program, and the species with the highest sequence similarity was identified.

### In vitro antibacterial activity assays of BPEO

The antibacterial activity of BPEO against the isolated BA-producing bacteria was evaluated by measuring the diameter of the inhibition zone, the MIC and the MBC. Specifically, the Oxford cup method was employed to determine the inhibition zone diameter of BPEO and pepper oleoresin, with an equal volume of distilled water as the negative control. Plates were incubated at 30 °C for 24 h, and inhibition zone diameters were measured. MIC and MBC were determined using the broth microdilution method^48^. BPEO was serially diluted in LB medium (0.05–100%) and cultured with bacterial suspension in a 96-well plate at 30 °C for 24 h. MIC was the lowest concentration without a color change after iodonitrotetrazolium chloride (INT) staining. For MBC, cultures from unstained wells were transferred to fresh medium, incubated, re-stained, and the lowest concentration without growth was recorded.

### Quantification of amino acid decarboxylase

The effect of BPEO on microbial amino acid decarboxylase levels was measured using a commercially available ELISA kit (Shanghai Tongwei Industrial Co., Ltd., China) according to the manufacturer’s instructions. The assay is based on a double-antibody sandwich ELISA principle and quantitatively measures amino acid decarboxylase protein levels in bacterial samples. Bacterial cultures without BPEO were used as the control group. To minimize potential interference of BPEO with absorbance measurements at 450 nm, a blank medium containing an equivalent amount of BPEO but without bacterial cells was included as a reference control. The optical density values were measured at 450 nm and converted to amino acid decarboxylase concentrations according to the standard curve. According to the manufacturer, the ELISA kit specifically recognizes microorganism-derived amino acid decarboxylase with no significant cross-reactivity to structurally related proteins.

### Effect of BPEO on fish head spoilage during storage

The sensory panel comprised nine trained members (five females and four males). A 10-point descriptive scoring scale was employed to assess freshness-related sensory attributes of fish heads. Two attributes (eye appearance and muscle texture), which are commonly used indicators of freshness deterioration, were evaluated, and the detailed scoring criteria are provided in Table S6. Each panelist independently scored the eyes and muscles of each sample. Sensory scores for the two attributes were analyzed separately. Results are expressed as mean ± SD (*n* = 9), and statistical comparisons between treatments were performed for each attribute at each storage time point.

The pH was measured according to GB 5009.237-2016. In short, 2.0 g of minced fish head sample was mixed with 20 mL of distilled water, ultrasonicated for 5 min, then centrifuged (4000 rpm, 10 min). The supernatant pH was measured using a pH meter (BELL Analytical Instruments (Dalian) Co., Ltd., Liaoning Province, China). TVB-N content was measured following GB 5009.228-2016. Briefly, 10 g of minced fish head sample was homogenized in 100 mL of water and filtered. The filtrate was alkalized with MgO and distilled; the volatiles were trapped in boric acid and titrated with 0.01 mol/L HCl. TVB-N results were expressed as mg/100 g sample.

The TVC was determined according to GB 4789.2-2016 using the dilution plate count method. Selective counts for specific spoilage organisms were also conducted: *Pseudomonas* spp. on CFC agar (25 °C, 48 h), H₂S-producing bacteria on *Aeromonas* ampicillin agar (30 °C, 48 h), and *Aeromonas* spp. on iron agar (25 °C, 48 h).

### Metabolomic analysis

Untargeted metabolomic analysis was performed on fish head muscle (M) and mucus (N) samples from control (C) and BPEO-treated groups (T) at days 0, 6, and 10 days (groups codes: MC, MT, NC, and NT). Metabolites were extracted using cold 80% methanol, vortexed, incubated on ice for 5 min, and then centrifuged (12,000 rpm, 20 min, 4 °C). The supernatant was diluted to 53% methanol and centrifuged again to remove any precipitate^49^.

LC-MS analysis utilized an ExionLC™ AD system (SCIEX) in conjunction with a QTRAP^®^ 6500 + mass spectrometer (SCIEX). Samples were injected onto an Xselect HSS T3 column (2.1 × 150 mm, 2.5 μm) using a 20-min linear gradient at a flow rate of 0.4 mL/min for the positive/negative polarity mode. The mobile phases consisted of eluent A (0.1% formic acid-water) and eluent B (0.1% formic acid-acetonitrile). The solvent gradient was set as follows: 2% B for 0–2 min, 2–100% B for 2–15 min, 100% B for 15–17 min, 100–2% B for 17–20 min. The mass spectrometer was operated in both positive and negative electrospray ionization modes. Data acquisition used multiple reaction monitoring with optimized parameters for each metabolite. Peak integration and correction were processed using SCIEX OS Version 1.4 software.

### Statistical analysis

The results were presented as mean ± SD. Each assay was conducted with at least three replicates, while the MIC, MBC, and amino acid decarboxylase were measured with six independent replicates. Multivariate analyses of metabolomic data were conducted in metaX. Data visualization was performed using the R package ggplot2 and GraphPad Prism 9.0.

### Ethical statement

Sensory evaluation procedures involving human participants were approved by the Ethics Committee of Changsha University of Science and Technology (No. CSUST-MEC-24025). All participants participated voluntarily and provided informed consent prior to the study.

## Supplementary information

41538_2026_871_MOESM1_ESM

## Data Availability

All data supporting the findings of this study are included in this article, except for the raw metabolomics data, which are not publicly available due to their large file size but are available from the corresponding authors upon reasonable request.
