# Supplementary material for: Preservation of bighead carp heads using black pepper essential oil: biogenic amine inhibition and metabolomic insights
Source: NPJ Sci Food. 2026 Apr 30;10:215. doi: 10.1038/s41538-026-00871-y (PMC13338441; doi:10.1038/s41538-026-00871-y)
Supplement: Supplementary file 1 — 41538_2026_871_MOESM1_ESM [file 41538_2026_871_MOESM1_ESM.doc]

**Table S1 Rescreening results of characteristic biogenic amine (BA)-producing bacteria isolated from bighead carp heads.**

| Strain number | Arginine | Ornithine | Phenylalanine | Histidine | Tryptophan | Lysine |
| --- | --- | --- | --- | --- | --- | --- |
| A1 | + | + + + | − | − | − | + + + |
| A2 | − | + + + | + | − | − | + + + |
| A3 | + + | + + + | − | − | − | + + + |
| A4 | + + | + + + | − | − | − | + + + |
| A5 | + + | + + + | − | − | − | + + + |
| A6 | + + | + + + | − | − | − | + + + |
| A7 | + + | + + + | − | − | − | + + + |
| A8 | + + | + + + | − | − | − | + + + |
| A9 | + + | + + + | − | − | − | + + + |
| A10 | − | + + + | − | − | − | + + + |
| A11 | + + + | + + + | − | − | − | + + + |
| A12 | + + + | + + + | − | − | − | + + + |
| O1 | + + + | + + + | − | − | − | + + + |
| O2 | + + | + + + | − | − | − | + + + |
| O3 | + | + + + | − | − | − | + + + |
| O4 | + + | + + + | − | − | − | + + + |
| O5 | + | + + + | − | − | − | + + + |
| P1 | + + + | + + + | − | − | − | + + + |
| P2 | + + | + + + | − | − | − | + + + |
| P3 | + + + | + + + | − | − | − | + + + |
| P4 | − | + + + | − | − | − | + + + |
| T1 | + + + | − | − | − | − | − |
| T2 | + + | + + | − | − | − | − |
| T3 | + + + | + + | − | − | − | − |
| T4 | + + + | + + | − | − | − | − |
| T5 | − | − | − | − | − | − |
| T6 | + | + + + | − | − | − | + |
| T7 | + | − | − | − | − | − |
| T8 | − | + + + | − | − | − | − |
| T9 | − | + + + | − | − | − | − |
| L1 | + + + | + + | − | − | − | − |
| L2 | + + + | − | − | − | − | − |
| L3 | + + + | + + | − | − | − | − |
| L4 | − | − | − | − | − | + |
| L5 | + + + | − | − | − | − | + |
| L6 | + + | + | − | − | − | − |
| L7 | + + + | + + | − | − | − | − |
| L8 | − | − | − | − | − | − |
| L9 | + + + | + + | − | − | − | − |
| L10 | + + + | + + | − | − | − | − |
| L11 | − | − | − | − | − | − |
| L12 | + + + | + + | − | − | − | − |
| L13 | − | + + | − | − | − | − |
| L14 | + + + | + + | − | − | − | − |
| L15 | + + + | − | − | − | − | − |
| L16 | + + + | − | − | − | − | + |

Note: “+” indicates a positive result after 48 h, with a larger number of “+” symbols representing a more pronounced color change.

**Table S2 Identification results of BA-producing bacteria isolated from bighead carp heads.**

| Strain number | DNA identification results | Identities |
| --- | --- | --- |
| P3 | *Pseudomonas lundensis* | 99.22% |
| O1 | *Obesumbacterium proteus* | 99.29% |
| A11 | *Hafnia paralvei* | 97.10% |
| A12 | *Hafnia alvei* | 98.59% |
| T3/L7 | *Pseudomonas veronii* | 99.65% / 99.57% |
| L3/L12/L14 | *Pseudomonas fildesensis* | 99.86% / 99.42% / 99.86% |

**Table S3 Shared differential metabolites in fish muscle samples following BPEO treatment.**

| No. | Compound | Folds Change | |
| --- | --- | --- | --- |
|  |  | 6 d | 10 d |
| Carbohydrates | | | |
| 1 | 1-Aminocyclopropane-1-carboxylic acid | 1.18 | 1.18 |
| 2 | alpha-D-Galactose 1-phosphate | 0.68 | 0.95 |
| 3 | alpha-Ketocaproic acid | 1.75 | 1.02 |
| 4 | Betaine | 1.81 | 0.70 |
| 5 | Carnosine | 0.57 | 1.44 |
| 6 | D-Glucarate | 1.42 | 0.91 |
| 7 | Galactaric acid | 1.60 | 0.92 |
| 8 | Histamine | 0.87 | 0.75 |
| 9 | Homogentisic Acid | 2.89 | 0.07 |
| 10 | Hydrocortisone | 2.08 | 1.42 |
| 11 | Hydroxyglutaric acid | 1.37 | 0.44 |
| 12 | Hypotaurine | 1.62 | 1.35 |
| 13 | L-2-Aminoadipic acid | 1.71 | 0.90 |
| 14 | N,N-Dimethylglycine | 1.25 | 1.49 |
| 15 | O-Acetyl-L-homoserine | 1.42 | 1.14 |
| 16 | Pantothenic acid | 1.56 | 0.84 |
| 17 | Sphinganine | 1.24 | 1.32 |
| 18 | Uric Acid | 1.89 | 0.48 |
| 19 | N-acetyl-glutamate | 1.25 | 1.55 |
| 20 | N-Acetyl-L-glutamic acid | 0.33 | 1.34 |
| Lipids | | | |
| 21 | 3-Methyl-2-oxobutanoic acid | 1.69 | 1.00 |
| 22 | 4-Aminobutyric acid | 0.66 | 0.76 |
| 23 | 5'-Deoxy-5'-(methylthio)adenosine | 0.17 | 1.39 |
| 24 | Anthranilic acid | 1.46 | 0.55 |
| 25 | Choline | 1.31 | 1.45 |
| 26 | Creatine | 1.21 | 1.09 |
| 27 | D-Erythrose 4-phosphate | 0.68 | 1.05 |
| 28 | D-Gluconic acid | 1.68 | 0.98 |
| 29 | D-Mannose 6-phosphate | 0.59 | 1.05 |
| 30 | Guanine | 1.72 | 1.34 |
| 31 | Hydroxypyruvic acid | 5.15 | 0.72 |
| 32 | L-Carnitine | 1.37 | 1.11 |
| 33 | Nicotinic Acid | 2.84 | 1.05 |
| 34 | Taurine | 1.44 | 1.17 |
| Nucleic acids | | | |
| 35 | (S)-2-Aminobutanoate | 1.38 | 1.38 |
| 36 | D-2-Aminobutyric acid | 1.42 | 1.27 |
| 37 | Glu-Glu | 2.45 | 0.14 |
| 38 | Glycyl-L-leucine | 0.54 | 0.17 |
| 39 | Glycylglycine | 0.43 | 1.36 |
| 40 | Homoarginine | 2.41 | 1.62 |
| 41 | Isonicotinamide | 0.74 | 1.83 |
| 42 | Methylguanidine | 1.32 | 1.16 |
| Steroids | | | |
| 43 | 3-Methylindole | 0.86 | 1.01 |
| 44 | Calcium D-Panthotenate | 1.49 | 0.89 |
| 45 | Dimethyl phthalate | 1.50 | 1.85 |
| 46 | L-allo-Isoleucine | 1.12 | 0.96 |
| 47 | Oleamide | 0.83 | 0.64 |
| 48 | Palmitoleic acid | 0.64 | 0.77 |
| 49 | Picolinic acid | 2.47 | 1.05 |
| 50 | Succinic anhydride | 1.50 | 0.71 |
| Peptides | | | |
| 51 | 1,4-Naphthoquinone | 1.22 | 1.01 |
| 52 | D-Fructose 1,6-bisphosphate | 0.28 | 0.99 |
| 53 | gamma-Glutamylglutamic acid | 0.44 | 0.25 |
| 54 | N,N,N-trimethyllysine | 0.33 | 1.28 |
| 55 | Sedoheptulose 7-phosphate | 0.51 | 0.96 |
| 56 | N-Acetyl-L-methionine | 0.53 | 0.50 |
| Vitamins and Cofactors | | | |
| 57 | D-3-Phenyllactic acid | 0.53 | 0.21 |
| 58 | Ectoine | 0.52 | 3.93 |
| 59 | Ergothioneine | 0.49 | 0.92 |
| 60 | Hydantoin-5-propionic acid | 5.77 | 0.75 |
| 61 | Isonicotinic acid | 1.43 | 1.20 |
| 62 | S-Sulfo-L-cysteine | 0.14 | 1.15 |
| Organic acids | | | |
| 63 | D-Fructose 6-phosphate | 0.57 | 1.08 |
| 64 | D-Glucose 6-phosphate | 0.60 | 1.15 |
| 65 | Glycine | 1.19 | 1.11 |
| 66 | Pyridoxal 5-phosphate | 2.31 | 0.84 |
| Uncategorized | | | |
| 67 | 2-Furoylglycine | 0.75 | 1.05 |
| 68 | 2-Hydroxy-6-Aminopurine | 1.53 | 1.26 |
| 69 | 2-Phenylglycine | 1.56 | 1.24 |
| 70 | 3-Hydroxy-hexadecanoyl carnitine | 0.21 | 3.30 |
| 71 | 5-Hydroxylysine | 0.69 | 0.76 |
| 72 | alpha-Nicotinamide adenine dinucleotide | 16.17 | 0.35 |
| 73 | Arachidonoylcarnitine | 0.43 | 1.52 |
| 74 | Choline Hydroxide | 1.16 | 1.54 |
| 75 | Dimethyl fumarate | 1.29 | 0.95 |
| 76 | Dl-3-Hydroxynorvaline | 1.75 | 0.91 |
| 77 | Dl-Indole-3-lactic acid | 0.85 | 0.94 |
| 78 | Dl-Norleucine | 1.59 | 0.62 |
| 79 | Dodecanoylcarnitine | 0.53 | 3.86 |
| 80 | Gly-Phe | 0.10 | 0.61 |
| 81 | Guanosine | 1.63 | 1.22 |
| 82 | H-Gly-Pro-OH | 0.24 | 1.11 |
| 83 | Homotaurine | 1.93 | 1.68 |
| 84 | Isobutyryl carnitine | 2.97 | 0.84 |
| 85 | L-arginino-succinate | 0.41 | 0.74 |
| 86 | Propionylcholine | 1.57 | 1.45 |
| 87 | S-Methyl-5'-thioadenosine | 0.18 | 1.24 |
| 88 | Trimethyllysine | 0.33 | 1.36 |
| 89 | Linoleoylcarnitine | 0.27 | 5.87 |
| 90 | Lysopc 20:4 | 0.55 | 1.39 |
| 91 | Methyl nicotinate | 607.79 | 330.33 |
| 92 | N-Acetylalanine | 0.56 | 0.52 |
| 93 | N-Acetylglycine | 1.61 | 0.58 |

Note: Red indicates differential metabolites upregulated compared to the control group, while green indicates differential metabolites downregulated compared to the control group.

**Table S4 Shared differential metabolites in mucus samples following BPEO treatment.**

| No. | Compound | Folds Change | |
| --- | --- | --- | --- |
|  |  | 6 d | 10 d |
| Carbohydrates | | | |
| 1 | 3-Phosphoglyceric acid | 0.15 | 2.87 |
| 2 | 4-Hydroxyphenylpyruvate | 1.40 | 2.14 |
| 3 | alpha-Ketocaproic acid | 0.28 | 1.32 |
| 4 | Aminomalonic acid | 1.58 | 1.67 |
| 5 | Betaine | 2.90 | 0.59 |
| 6 | Cholic acid | 4.50 | 0.19 |
| 7 | Cortisone | 4.81 | 4.19 |
| 8 | D-Galactonic acid | 0.28 | 12.58 |
| 9 | D-Glutamine | 0.75 | 1.26 |
| 10 | L-2-Aminoadipic acid | 0.29 | 0.49 |
| 11 | L-Hydroxyproline | 0.72 | 1.25 |
| 12 | O-Phospho-L-serine | 0.16 | 3.30 |
| 13 | Phosphocholine | 0.30 | 0.38 |
| 14 | Pipecolic acid | 0.67 | 1.26 |
| 15 | Sedoheptulose 1,7-bisphosphate | 2.92 | 0.72 |
| 16 | Sepiapterin | 0.50 | 0.34 |
| 17 | Shikimic Acid | 3.57 | 1.93 |
| 18 | Xanthine | 1.59 | 0.40 |
| Lipids | | | |
| 19 | 4-Aminobutyric acid | 2.30 | 1.95 |
| 20 | 4-Hydroxybenzoic acid | 1.75 | 1.54 |
| 21 | acetoacetate | 0.41 | 0.72 |
| 22 | Adenylyl Sulfate | 0.26 | 0.22 |
| 23 | CDP | 0.25 | 0.14 |
| 24 | D-Gluconic acid | 0.34 | 13.75 |
| 25 | D-Glucosamine 6-phosphate | 0.32 | 0.24 |
| 26 | D-Glyceraldehyde 3-phosphate | 1.27 | 1.55 |
| 27 | D-Mannose 6-phosphate | 0.34 | 2.47 |
| 28 | Hydroxypyruvic acid | 2.63 | 2.00 |
| Peptides | | | |
| 29 | 1-Methylhistamine | 1.32 | 1.58 |
| 30 | 3-Ureidopropionate | 0.61 | 0.51 |
| 31 | Cycloleucine | 0.74 | 0.86 |
| 32 | Glycerol-3-phosphate | 0.39 | 0.23 |
| 33 | Hydroxyphenyllactic acid | 1.62 | 1.94 |
| 34 | L-Octanoylcarnitine | 0.34 | 0.17 |
| 35 | Methyl beta-D-galactopyranoside | 2.50 | 2.31 |
| 36 | N-Acetylglucosamine 1-phosphate | 0.42 | 0.23 |
| 37 | Pimelic acid | 1.62 | 2.11 |
| 38 | Taurocholic acid | 2.32 | 2.89 |
| Organic acids | | | |
| 39 | Adenosine 5'-Diphosphate | 0.38 | 0.25 |
| 40 | D-Fructose 6-phosphate | 0.38 | 2.43 |
| 41 | D-Glucopyranose | 1.62 | 1.95 |
| 42 | D-Glucose 6-phosphate | 0.38 | 2.94 |
| 43 | L-Ornithine | 0.38 | 2.93 |
| 44 | Phosphopyruvic acid | 0.21 | 4.38 |
| 45 | UDP-galactose | 0.15 | 0.31 |
| 46 | UDP-N-acetylglucosamine | 0.28 | 0.30 |
| Steroids | | | |
| 47 | Dimethyl phthalate | 3.16 | 3.79 |
| 48 | Hexadecanedioate | 1.38 | 1.60 |
| 49 | N7-Methylguanosine | 0.49 | 0.29 |
| 50 | Octanedioic acid | 1.20 | 1.69 |
| 51 | Palmitoleic acid | 0.76 | 0.63 |
| 52 | Raubasine | 2.56 | 2.34 |
| 53 | Succinic anhydride | 2.71 | 2.48 |
| 54 | Tiglic acid | 2.72 | 2.42 |
| Nucleic acids | | | |
| 55 | D-2-Aminobutyric acid | 1.56 | 1.13 |
| 56 | Decanoic acid | 1.73 | 2.14 |
| 57 | Mefenamic acid | 1.16 | 1.42 |
| 58 | Phosphocreatine | 2.14 | 24.05 |
| Vitamins and Cofactors | | | |
| 59 | 2-Hydroxybutyric acid | 1.97 | 1.49 |
| 60 | 20-Carboxy-Leukotriene B4 | 0.63 | 0.71 |
| 61 | 4-Hydroxy-L-Glutamic Acid | 1.19 | 1.43 |
| 62 | Hydantoin-5-propionic acid | 2.79 | 2.43 |
| Uncategorized | | | |
| 63 | 13-Hpotre(R) | 0.50 | 1.68 |
| 64 | 2-C-Methyl-D-erythritol 2,4-cyclodiphosphate | 0.49 | 3.01 |
| 65 | 2-deoxyglucose-6-phosphate | 0.46 | 0.36 |
| 66 | 2-Phosphoglyceric acid | 0.23 | 2.44 |
| 67 | 3-Methyladipic acid | 1.58 | 2.00 |
| 68 | 4-aminobutyrate | 2.25 | 1.99 |
| 69 | 4-Hydroxyphenyllactate | 1.72 | 1.91 |
| 70 | 7-Ketocholesterol | 2.70 | 6.96 |
| 71 | Adipamide | 8.73 | 0.30 |
| 72 | beta-Alanine methyl ester | 2.50 | 2.02 |
| 73 | Choline Glycerophosphate | 0.41 | 0.30 |
| 74 | D-2-Aminoadipic acid | 1.28 | 1.44 |
| 75 | Dl-Norleucine | 2.91 | 0.58 |
| 76 | Gamma-Glu-Leu | 0.34 | 0.13 |
| 77 | hydroxyphenylpyruvate | 0.11 | 0.26 |
| 78 | L-arginino-succinate | 0.39 | 1.80 |
| 79 | Lysine Butyrate | 4.21 | 0.44 |
| 80 | Methyl alpha-D-glucopyranoside | 2.36 | 2.53 |
| 81 | N-(5-Aminopentyl)acetamide | 5.62 | 0.37 |
| 82 | N-Acetylglycine | 2.74 | 0.53 |
| 83 | N',N""",N"""'-P-Coumaroyl-Cinnamoyl-Caffeoyl Spermidine | 2.11 | 5.00 |
| 84 | N6-Succinyl Adenosine | 0.29 | 0.64 |
| 85 | Tetraethylammonium fluoride dihydrate | 0.55 | 6.66 |
| 86 | Undecanedioic acid | 1.54 | 2.30 |
| 87 | Uridine 5'-Diphospho-N-Acetylgalactosamine | 0.18 | 0.27 |
| 88 | Val-Ser | 0.54 | 0.55 |

Note: Red indicates differential metabolites upregulated compared to the control group, while green indicates differential metabolites downregulated compared to the control group.

**Table S5 PCR amplification reaction system**

| Reagents | Volume (μL) |
| --- | --- |
| Genomic DNA（20 ng/μL） | 1.0 |
| 10 × Buffer（including 2.5 mM Mg^2+^） | 5.0 |
| Taq polymerase（5 U/μL） | 1.0 |
| dNTP（10 mM） | 1.0 |
| 27F primer（10 μM） | 1.5 |
| 1492R primer（10 μM） | 1.5 |
| ddH_2_O | 39.0 |
| Total volume | 50.0 |

**Table S6 Sensory evaluation criteria**

| Attribute | Scoring criteria | Score |
| --- | --- | --- |
| Eyes | Transparent and bright, not cloudy, eye muscles are elastic | ≥ 8 |
|  | Corneal opacity, reduced eye muscle elasticity | 6–8 |
|  | Sunken and cloudy eyes, eye socket filled with white haze, iris hemorrhage | < 6 |
| Muscles | Firm and elastic muscle near the incision, cut surface is shiny and bright | ≥ 8 |
|  | Reduced elasticity of muscle near the incision, soft texture upon finger pressure, dull cut surface | 6–8 |
|  | Loss of elasticity in muscle near the incision, muscle easily separated, fishy odor present | < 6 |


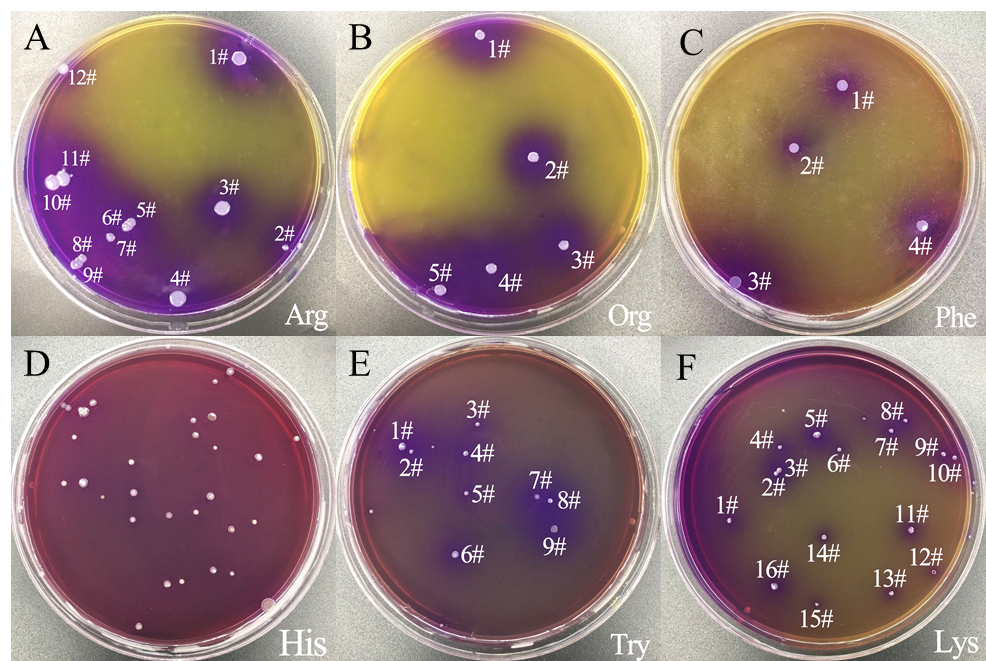


# **Fig. S1**. Screening of BA-producing bacteria isolated from bighead carp head during superchilling storage. BAs chromogenic medium supplemented with arginine (A), ornithine (B), phenylalanine (C), histidine (D), tryptophan (E) and lysine (F) was used for the screening.


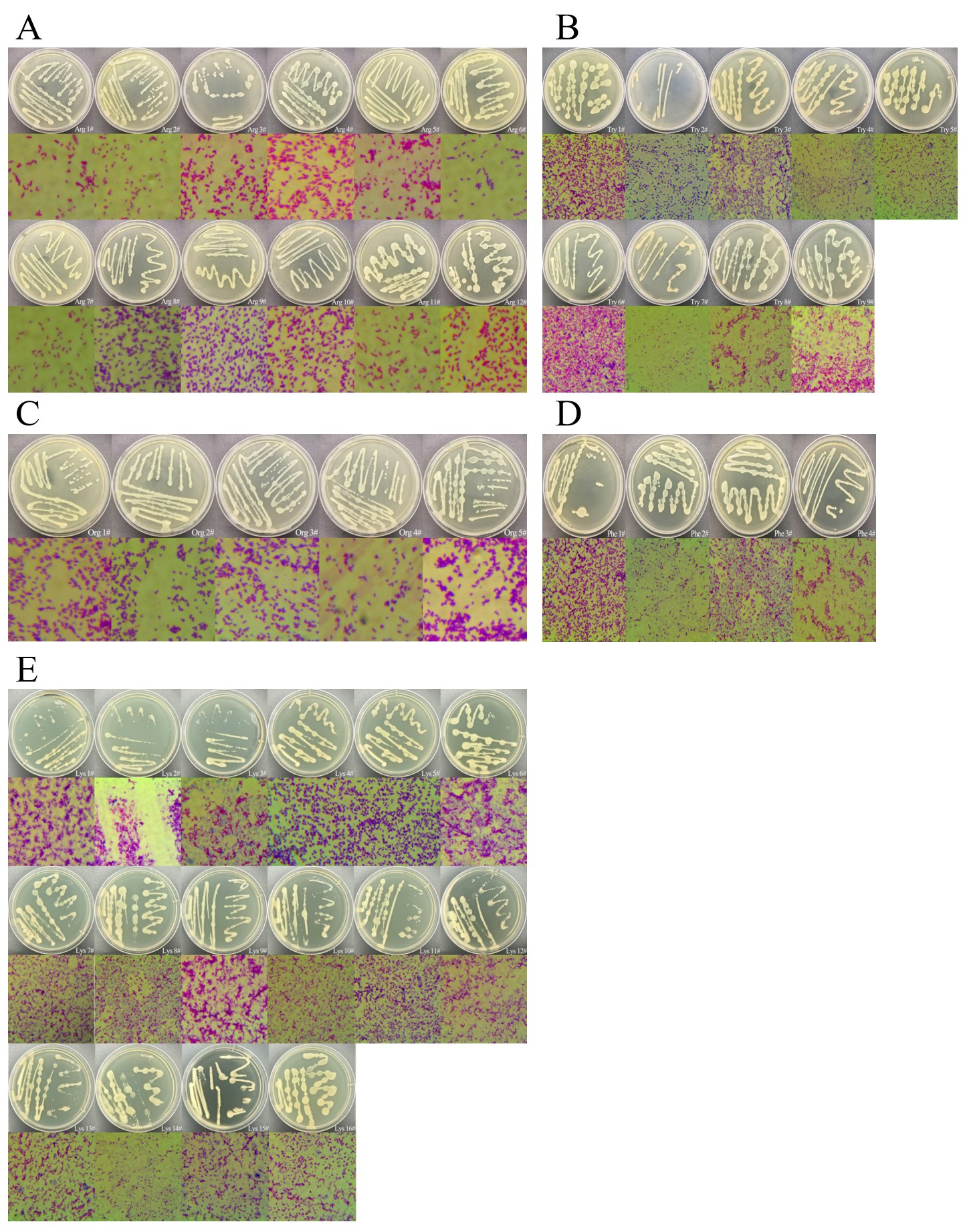
**Fig. S2**. Purification and gram staining of BA-producing bacteria screened with different amino acid precursors: (A) arginine , (B) tryptophan, (C) ornithine, (D) phenylalanine and (E) lysine.


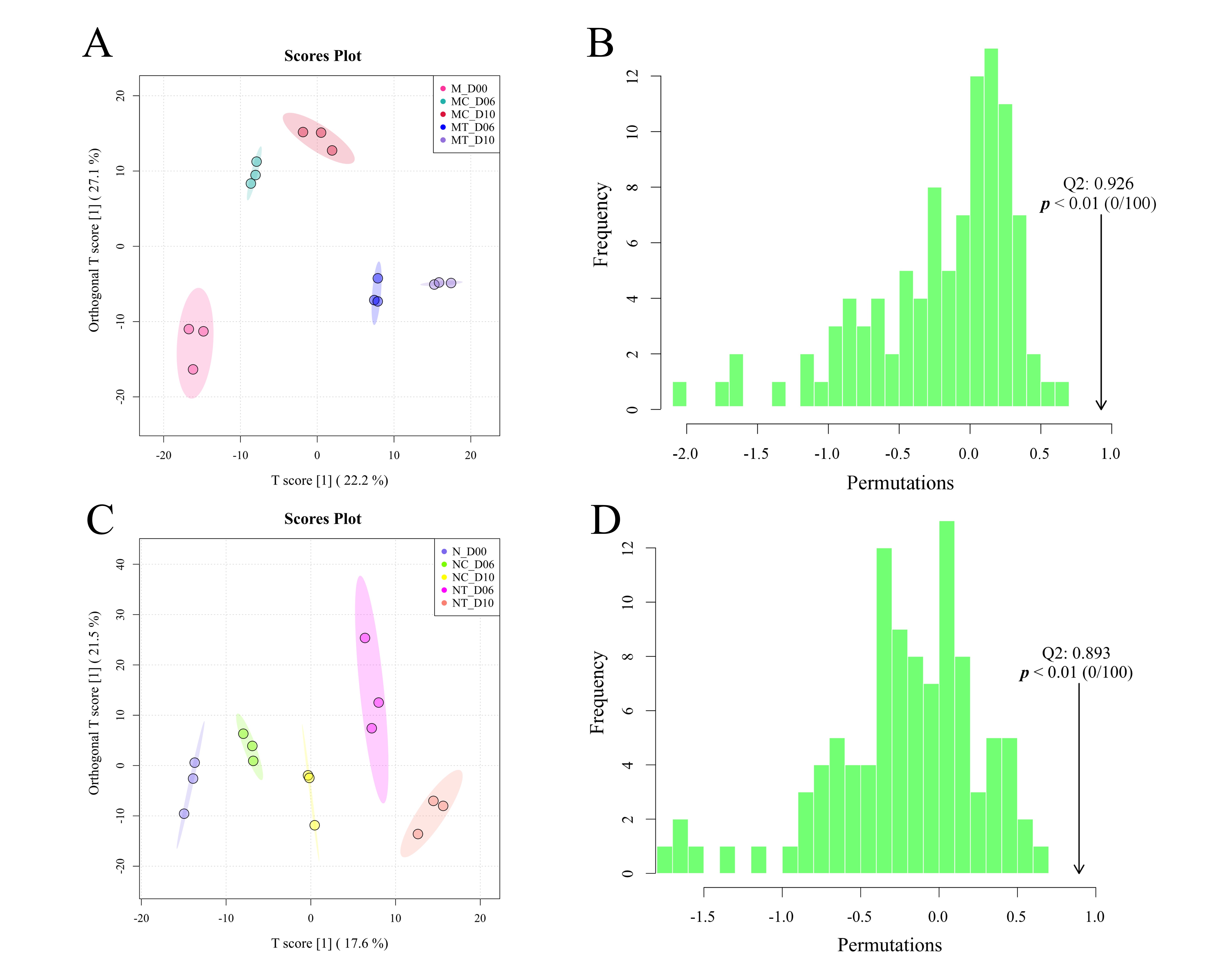
**Fig. S3.** Orthogonal partial least squares discrimination analysis (OPLS-DA) of fish flesh and mucus samples. Score plot of fish meat (A) and mucus (C). Cross-validation of the corresponding OPLS-DA models for fish meat (B) and mucus (D).


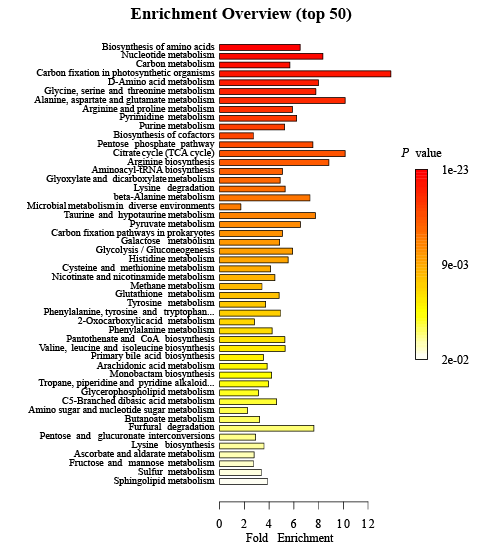

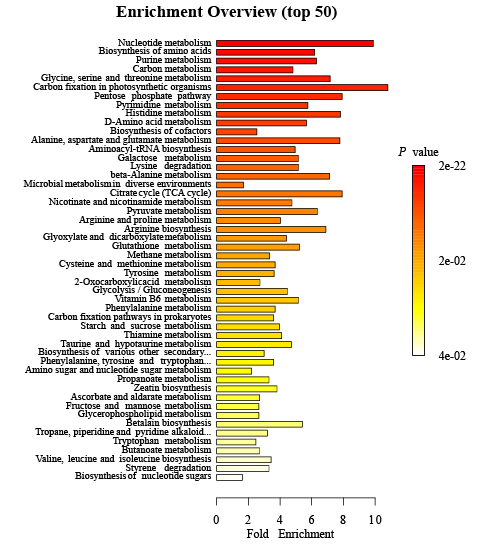


A

B

**Fig. S4.** Enrichment analysis of major differential metabolites in fish meat (A) and mucus (B) samples.


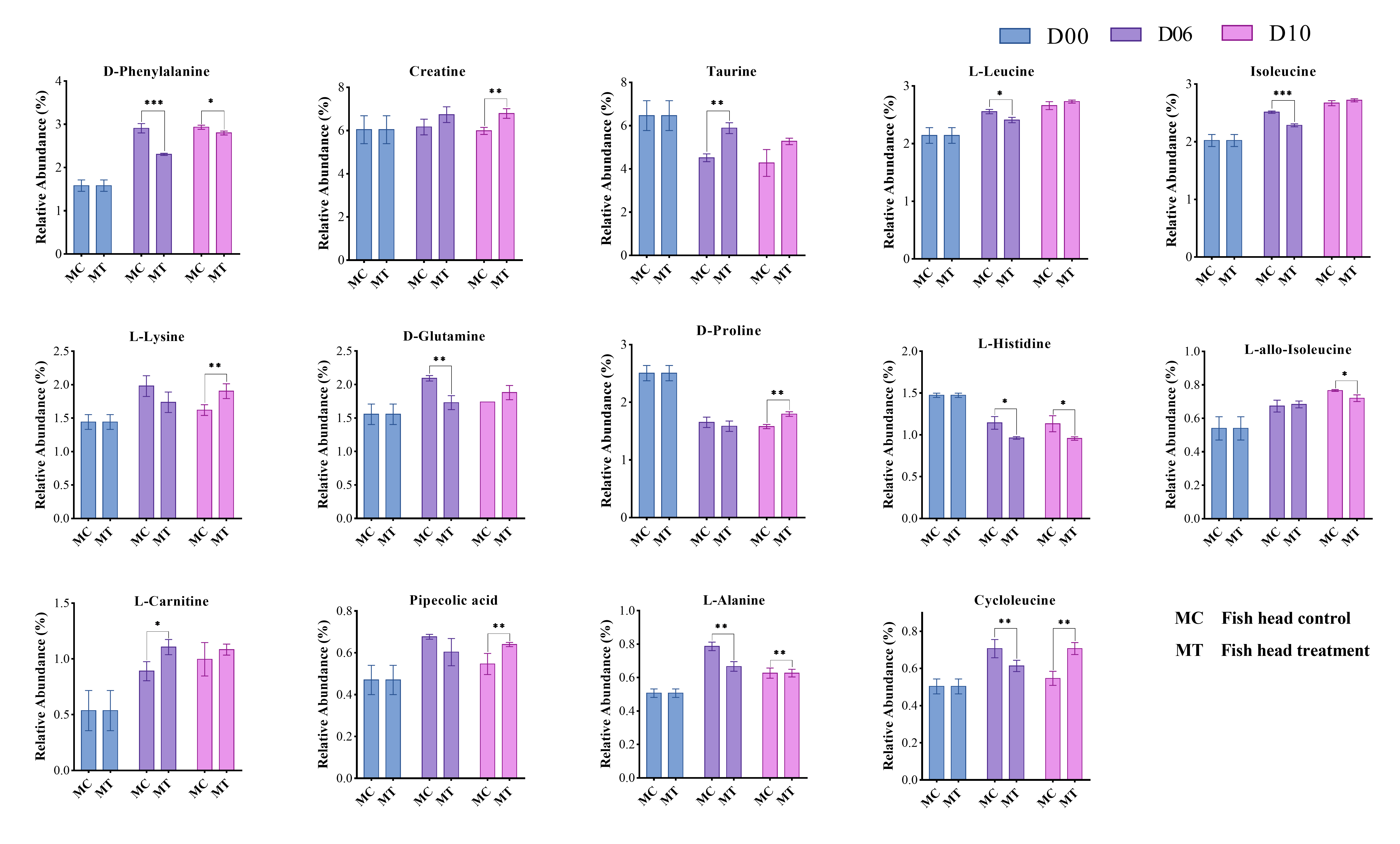
**Fig. S5.** Relative abundance of differential metabolites of major amino acids in fish meat after BPEO treatment.


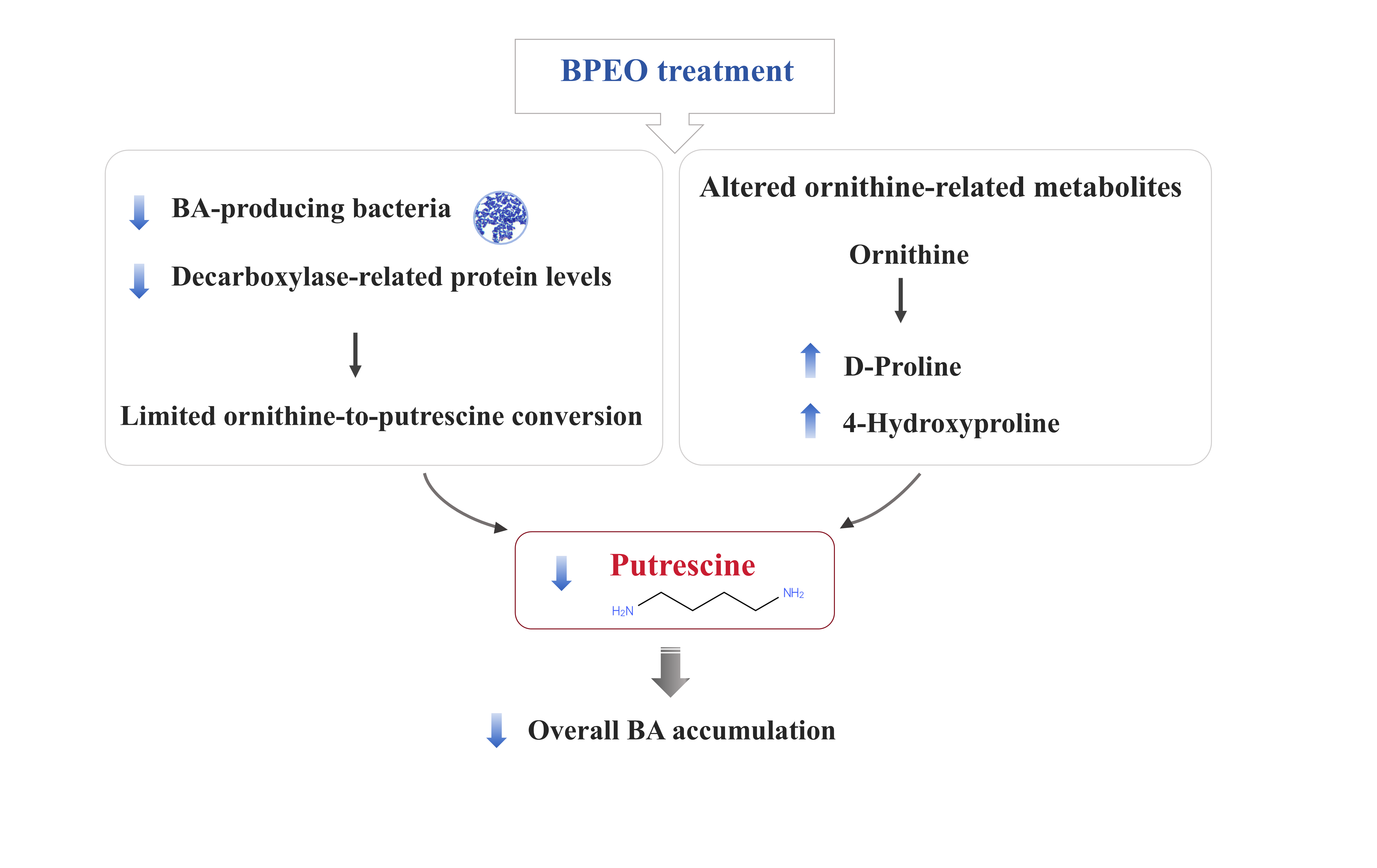
**Fig. S6.** Simplified schematic illustrating associations between BPEO treatment, microbial changes, and putrescine reduction.
